# Associations of *LEP*, *CRH*, *ICAM-1*, and LINE-1 methylation, measured in saliva, with waist circumference, body mass index, and percent body fat in mid-childhood

**DOI:** 10.1186/s13148-017-0327-5

**Published:** 2017-03-29

**Authors:** Jocelyn Dunstan, Joseph P. Bressler, Timothy H. Moran, Jonathan S. Pollak, Annemarie G. Hirsch, Lisa Bailey-Davis, Thomas A. Glass, Brian S. Schwartz

**Affiliations:** 1 Department of Epidemiology, Johns Hopkins Bloomberg School of Public Health, Baltimore, MD USA; 2 Department of Environmental Health and Engineering, Johns Hopkins Bloomberg School of Public Health, Baltimore, MD USA; 3 Department of Psychiatry and Behavioral Sciences, Johns Hopkins School of Medicine, Baltimore, MD USA; 4 Department of Epidemiology and Health Services Research, Geisinger Health System, Danville, PA USA

**Keywords:** Obesity, Epigenetics, DNA methylation, Sex differences, *LEP*, *CRH*, *ICAM-1*, LINE-1

## Abstract

**Background:**

Genetics explains a small proportion of variance in body mass index at the population level. Epigenetics, commonly measured by gene methylation, holds promise for understanding obesity risk factors and mechanisms.

**Methods:**

Participants were 431 adolescents aged 10–15 years. BMI z-score, waist circumference z-score, and percent body fat were measured. Saliva samples were collected and methylation of promoter regions of four candidate genes or sequences (*LEP*, *ICAM-1*, *CRH*, and LINE-1) were measured in 3–4 CpG sites each. Linear regression was used to identify associations of methylation with obesity-related outcomes.

**Results:**

After adjusting for age, in sex-stratified analysis, the three obesity-related outcomes were negatively associated with *LEP* methylation in obese boys only. There were no associations of methylation of the other genes or sequences and the obesity-related outcomes.

**Conclusions:**

Our results are consistent with prior studies that reported sex differences in associations of obesity-related outcomes with *LEP* methylation, and also as would be expected in adipose tissue, the source of circulating leptin. The findings suggest that saliva might be an acceptable tissue for epigenetics studies in adolescents.

**Electronic supplementary material:**

The online version of this article (doi:10.1186/s13148-017-0327-5) contains supplementary material, which is available to authorized users.

## Background

Childhood obesity is a major public health concern worldwide [1]. Obese children and adolescents may experience physical and psychological consequences, such as increased risk for the development of diabetes and cardiovascular diseases as well as low self-esteem [2]. Childhood obesity often persists into adulthood [3], with associated increased risk of premature mortality [4].

In the USA, the prevalence of obesity in 2013–2014 for children and adolescents between 6–11 and 12–19 years of age was 19.6 and 20.6%, respectively [5]. The prevalence differed by race/ethnicity, with evidence of highest prevalence in the Hispanic/Latino population [5, 6]. Since genetic variation explains only a small proportion of the variation in BMI [7, 8], a growing number of studies have investigated the role of epigenetic factors. Gene expression is partially regulated by epigenetic mechanisms, which are changes that are not due to alterations of the DNA sequence [9]. Epigenetic mechanisms include DNA methylation, histone modification, and DNA-binding proteins [10].

Epigenetic changes in genes related to stress and appetite have been studied as predictors of satiety, appetite, and obesity [11]. In epidemiological studies, DNA methylation is the most frequent epigenetic mechanism studied because it can be measured in surrogate tissues in large populations in quantitative assays. The use of surrogate tissues requires evaluation like that presented herein. DNA methylation occurs primarily at cytosine within cytosine-guanine dinucleotides (CpG), and it affects gene expression by modifying the degree to which the DNA is accessible to promoters or suppressors [12].

This research evaluated associations between DNA methylation, measured in saliva samples, and anthropometric and body composition measurements (hereafter called “obesity-related measures”) in 431 adolescents between 10 and 15 years of age. Associations were also examined in relation to medical information in electronic health records (EHR), dietary and physical activity behavioral questionnaires from the child, and parental BMI. We measured methylation in 3–4 CpG sites in the promoter regions of three candidate genes selected on the basis of different biological pathways that are involved in obesity: leptin (*LEP*, associated with appetite and fat metabolism [13]), corticotropin-releasing hormone (*CRH*, stress and appetite [14]), and intracellular adhesion molecule (*ICAM-1*, inflammation [15]). In addition, methylation in the LINE-1 gene sequence was used to evaluate whether methylation in candidate genes evidenced specificity.

## Methods

### Participants and design

Subjects were identified, recruited, and enrolled during the summer months of 2013 and 2014. First, target communities were identified according to three criteria: (1) located within the Geisinger Clinic’s 45-county catchment area; (2) had at least 75 primary care patients aged 10–15 years, the target age range for the study; and (3) it fell into one of the four cells of the community selection table, defined by having: (a) high or low proportions of overweight (BMI for age/sex percentile ≥85th) and obese (BMI for age/sex percentile ≥95th) children and (b) community environments considered obesogenic or obesoprotective based on community socioeconomic deprivation, population density, and physical activity diversity [16]. For the third criterion, environmental variables and community overweight/obesity prevalence were each first divided into quintiles along a gradient of obesogenic to obesoprotective environments and high to low overweight/obesity prevalence. Then, communities were selected in the first or fifth quintile for each in four strata: (1) obesogenic environment, high prevalence; (2)obesogenic environment, low prevalence; (3) obesoprotective environment, high prevalence; and (4) obesoprotective environment, low prevalence.

Children and adolescents (hereafter “children”) were first identified using EHR to obtain age and address information. A pre-notification letter was next sent that provided the parent the option of opting out of the study. If this was not returned within 10 days, the household was next contacted by phone to enroll the child and one parent in the study. If the household agreed to participate, a home visit was scheduled. At each household visit, one parent/guardian and one child between 10 and 15 years of age were enrolled. A total of 431 dyads were enrolled from 28 communities that included 9 boroughs, 11 townships, and 8 census tracts ranging (median) from 7 to 28 (14.5) enrolled children per community.

Each household received a $30 gift card for the time and effort. In 2013, 210 child-parent dyads were enrolled (22.2% participation rate) and in 2014, 222 additional dyads participated (14.8% participation rate). The most common reasons for lack of participation included passive refusal after 18 phone calls, active refusal after telephone contact, and incorrect telephone number [17]. The study was approved by the Institutional Review Boards at both the Geisinger Health System and the Johns Hopkins Bloomberg School of Public Health.

### Data collection

During the in-home visit, trained research staff measured height using a portable stadiometer (model seca 213, seca North America, Inc.); weight and percent body fat (PBF, three times) with a calibrated portable digital scale with tetrapolar bioelectrical impedance analysis (model TBF-300A, TANITA Corporation of America, Inc., Arlington Heights, IL); and waist circumference (WC, three times) with a measuring tape. The measurements were taken following the NHANES anthropometric procedures manual [18]. Shoes and socks were removed prior to height and weight measurements, and children were asked to empty their pockets and remove heavy layers of clothing. The waist circumference was measured with one shirt layer and at the hip bone level. Participants were asked to cross their arms and place them at their opposite shoulders, and the measurement was taken during normal expiration.

Height and weight were used to estimate the body mass index (BMI, kg/m^2^). Age- and sex-specific BMI z-scores (hereafter BMI-z) were created based on national standards [19]. Similarly, measured WC mean values were used to create WC z-scores (WC-z) as previously reported [20]. Demographic information was obtained from a parent during the initial telephone contact. During the home visit, the child completed a self-administrated questionnaire about food and physical activity environments and habits, as previously reported [17]*.* Data from EHR included use of Medical Assistance for health insurance (as a proxy measure of low family socioeconomic status (SES)), medication history (e.g., antibiotics, stimulants, antidepressants, and anti-anxiety agents), and selected diagnoses (e.g., asthma, diabetes, food allergies, other allergies, and mental health [ICD-9 codes 290.x to 319.x]). These were created as ever vs. never variables and evaluated in regression models one at a time.

### Epigenetic measures

From each child, a saliva specimen was collected during the home visit using the Oragene-DISCOVER kit (OGR-500, DNA Genotek, Inc, Ontario, Canada). DNA bisulfite conversion was accomplished using EZ DNA Methylation Kits (Zymo Research) by following the manufacturer’s manual. Briefly, 0.5–1.0 μg of genomic DNA was first mixed with 5 μl of M-Dilution Buffer and incubated at 37 °C for 15 min and then mixed with 100 μl of CT Conversion Reagent. Mixtures were incubated in a thermocycler with 16 thermal cycles at 95 °C for 30 s and 50 °C for 1 h. Bisulfite-converted DNA samples were loaded onto 96-column plates provided in the kit for desulfonation and purification. The concentration of eluted DNA was measured using a NanoDrop 1000 spectrometer.

DNA methylation was quantitated using bisulfite-PCR and pyrosequencing using the following kits from Qiagen: ICAM1_02 for *ICAM-1*, CRH_01 for *CRH*, LEP_01 for *LEP*, and NOS3_02 for LINE-1. Sequencing was conducted using Pyromark Q24, and percent methylation was computed using the Pyromark Q24 Software v.2 in CpG mode. Details about the location of the measured CpG sites are in the online supporting information (Additional file 1: Table S1).

### Statistical analysis

The primary goal of the analysis was to evaluate associations of obesity-related measures (BMI-z, WC-z, and PBF) with DNA methylation at each CpG site and for the mean of that gene across sites while adjusting for potential confounding by age. Before performing the analysis, we considered the causal ordering of DNA methylation and obesity [21]. These are complex relationships that may involve bi-directional influences. However, we hypothesized that LINE-1, *CRH*, and *ICAM-1* were predominantly antecedent contributors to weight gain. Thus, for these loci, we modeled obesity as the outcome with methylation as the predictor. In contrast, for *LEP*, because obesity is known to influence leptin levels and triggers leptin resistance [21], we modeled *LEP* methylation as the outcome with obesity as the predictor. Analysis was performed using Stata 14 (StataCorp LP, College Station, TX).

Summary statistics were first examined for all obesity-related measures and for all DNA methylation by CpG site and as overall gene means, for all children combined, and then for boys and girls separately. Associations were evaluated using linear regression, first for all children combined, and then separately for boys and girls in a single model with a cross-product term between sex and the variable of interest. The model was adjusted for the child’s age (centered) and evaluated non-linearity by inclusion of a quadratic term for age. Linear regression beta coefficients, standard errors, and *p* values are reported.

Variables were also created from the child questionnaire, previously described [17] and evaluated in association with DNA methylation (three genes and one gene sequence) or obesity-related outcomes (as confounders of the relation with *LEP* methylation). The questions were about home environment and the child’s dietary and physical activity behaviors. Several questionnaire items were combined to create summary scores [22], as continuous variables if distributions were not highly skewed, or using tertiles if they were. Variables were only retained in the model if they had an associated *p* value <0.05 when included in the model and if the beta coefficient for the primary predictor variable changed by more than 10% when the new variable was included. BMI was modeled as a continuous variable and also as indicator variables for overweight (BMI-z between 85th and 95th percentile) and obesity (BMI-z over the 95th percentile).

Reported results are not corrected for multiple comparisons. Normality of residuals, homoscedasticity, independence (collinearity), and sensitivity to outliers were evaluated in the final model by examination of variance inflation factors and added variable plots. A *p* value <0.05 was considered statistically significant.

## Results

### Description of the study subjects

Of the 431 children included in the study, 210 were boys and 221 were girls. Their mean age was just under 13 years for boys and girls, 93% were Caucasian, 35% received Medical Assistance for health insurance, and sizable portions were overweight or obese (Table 1). The large majority of participating parents were mothers with mean BMI values approaching 30 kg/m^2^.

**Table 1:** Summary statistics of participating children and parents

	All	Males	Females
Children			
*N* (%)	431 (100)	210 (48.72)	221 (51.28)
Mean age, years (SD)	12.86 (1.70)	12.77 (1.64)	12.93 (1.75)
Race/ethnicity			
Caucasian (%)	395 (92.72)	187 (90.78)	208 (94.55)
Non-Caucasian (%)	31 (7.28)	19 (9.22)	12 (5.45)
Body mass index, kg/m^2^			
<85th percentile (%)	285 (65.67)	135 (64.29)	150 (66.96)
85th to 95th percentile (%)	71 (16.36)	32 (15.24)	39 (17.41)
≥95th percentile (%)	78 (17.97)	43 (20.48)	35 (15.62)
Waist circumference z-score (%)	0.48 (0.92)	0.55 (0.95)	0.41 (0.89)
Percent body fat (SD)^a^	22.96 (11.31)	19.73 (11.18)	26.01 (10.57)
Received medical assistance (%)	154 (35.48)	82 (39.05)	72 (32.14)
Parents			
*N* (%)	416 (100)	60 (14.42)	356 (85.58)
Mean age, years (SD)	42.62 (8.22)	47.30 (8.40)	41.83 (7.93)
Body mass index, kg/m^2^ (SD)	29.17 (7.48)	29.73 (5.78)	29.08 (7.73)

This table shows characteristics of our sample stratified by sex

^a^Percent body fat was available for only 430 children

Methylation levels at all CpG sites measured in the *CRH* and *ICAM-1* genes were low in both boys and girls with minimal variation (online supporting information, Additional file 2: Table S2). Mean methylation levels in LINE-1 were high for both boys and girls, and there were no differences by sex or obesity status. Methylation levels of *LEP* differed by CpG site; there were also significant differences between boys and girls (Additional file 2: Table S2).

### Associations of obesity-related measures and *LEP* methylation

**Fig. 1:**
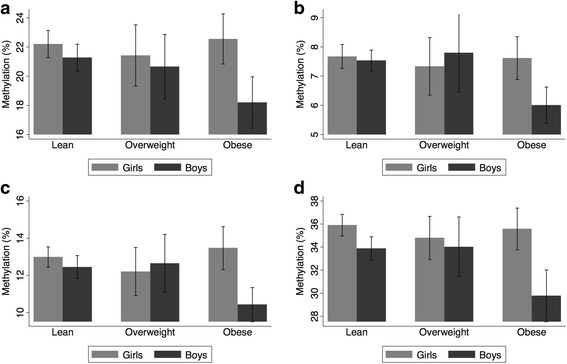
*LEP* methylation in each CpG site, by sex, for lean, overweight, and obese children. **a** CpG-1, **b** CpG-2, **c** CpG-3, and **d** CpG-4

Obesity was consistently and strongly associated with *LEP* methylation and that association varied by sex (Table 2 and Fig. 1). Parental BMI was associated with both *LEP* methylation and obesity-related outcomes and changed the primary association more than 10%; therefore, it was retained in all subsequent models. In contrast, inclusion of EHR or child questionnaire-derived variables in the models did not identify any as significant predictors of *LEP* methylation. These variables also did not change relations of any obesity-related measure with *LEP* methylation.

**Table 2:** Stratified analysis (boys and girls) of adjusted associations for leptin methylation at four CpG sites and for overall mean as outcome and the three obesity-related measures as primary predictors

Predictor	LEP methylation (dependent variable)														
	CpG-1			CpG-2			CpG-3			CpG-4			Mean		
	Boys β (SE)	Girls β (SE)	*P* ^§^	Boys β (SE)	Girls β (SE)	*P*	Boys β (SE)	Girls β (SE)	*P*	Boys β (SE)	Girls β (SE)	*P*	Boys β (SE)	Girls β (SE)	*P*
BMI-z	−0.765* (0.334)	0.429 (0.371)	0.02	−0.341** (0.146)	0.001 (0.162)	0.11	−0.651** (0.210)	0.286 0.234	0.003	−1.058** (0.364)	0.190 (0.405)	0.02	−0.704** (0.225)	0.227 (0.250)	0.01
WC-z	−1.064* (0.429)	0.443 (0.456)	0.01	−0.515** (0.187)	−0.079 (0.199)	0.10	−0.681* (0.272)	0.341 (0.289)	0.01	−1.235** (0.469)	0.273 (0.498)	0.03	−0.874** (0.289)	0.245 (0.307)	0.01
PBF	−0.089* (0.036)	0.057 (0.039)	0.01	−0.036* (0.016)	0.005 (0.017)	0.08	−0.062** (0.022)	0.030 (0.025)	0.01	−0.102* (0.039)	−0.009 (0.044)	0.11	−0.072** (0.024)	0.021 (0.027)	0.01

Ordinary least squares regression models were adjusted for age and parental BMI

*BMI-z* body mass index z-score, *WC-z* waist circumference z-score, *PBF* percent body fat

*P* values: **0.001 ≤ *P* < 0.01; *0.01 ≤ *P* < 0.05

^§^ *P* value for interaction term between leptin methylation and sex

The associations in the regression models were primarily driven by obese boys (Fig. 1). This was confirmed when the regression models were repeated in boys only, with indicators for overweight and obesity as the primary predictors (Table 3). The beta coefficients for obesity were negative and relatively large, indicating that obesity in boys was associated with lower *LEP* methylation levels.

**Table 3:** Adjusted associations for *LEP* methylation in boys with overweight and obese status as primary predictor

Obesity status (predictors)	*LEP* methylation in boys (dependent variable)				
	CpG-1 β (SE)	CpG-2 β (SE)	CpG-3 β (SE)	CpG-4 β (SE)	Mean β (SE)
Overweight	0.373 (1.140)	0.730 (0.495)	0.850 (0.719)	1.207 (1.243)	0.790 (0.768)
Obese	−2.822** (0.993)	−1.507*** (0.432)	−2.149*** (0.627)	−3.961*** (1.080)	−2.610*** (0.666)

Models adjusted for age and parental BMI

*P* values: ***P < 0.001; **0.001 ≤ *P* < 0.01; *0.01 ≤ *P* < 0.0

### Associations of *CRH*, *ICAM-1*, and LINE-1 methylation with obesity-related outcomes

Linear regression was next used to evaluate associations of methylation levels in *CRH*, *ICAM-1*, and LINE-1 in relation to the three obesity-related outcomes. There were no associations for *CRH* or LINE-1 with any obesity-related measure for either boys or girls (results not shown). For *ICAM-1*, methylation of CpG1 was inversely associated with PBF in boys; mean methylation was inversely associated with WC-z in boys; and the cross product *p* value between CpG1 and sex was significant for all three obesity-related outcomes, with opposite directions of associations for boys and girls (online supporting information, Additional file 3: Table S3, Additional file 4). Inclusion of EHR variables (e.g., SES, child medication history, child diagnoses), child questionnaire-derived variables, and parental BMI in these models did not substantively change associations or inferences.

## Discussion

This is the first large study to investigate associations between DNA methylation in candidate genes measured in saliva, related to appetite, stress, and inflammation, and obesity-related outcomes in children and adolescents. Three genes were chosen because they are important regulators of biological pathways involved in obesity: *CRH* (stress), *ICAM-1* (inflammation), and *LEP* (appetite). A strong association was found between increased methylation in the *LEP* promoter region in boys but not in girls. No associations were detected with methylation in the other genes, which had low absolute values with little variation, perhaps because of the CpG sites that were measured.

Several important confounding variables were evaluated, including child self-reported information on diet and physical activity, and medical history and medications from EHR data. None of these variables were associated with *LEP* methylation levels. Analysis of EHR medication data was of interest, for example, because we have previously reported that stimulants were associated with BMI trajectories in children [23], and we were interested to evaluate whether this association may be mediated through *LEP* methylation, as both circulating leptin and stimulants affect appetite [7, 24].

Parental BMI was associated with both *LEP* methylation and the obesity-related measures. There is consistent with prior evidence that parental BMI should be strongly associated with child BMI [3]. Most of the parents in the study were female (86%), and some studies have looked at epigenetic changes passed by mothers to children, for example by measuring *LEP* methylation in placental tissues [25]. We only had information about parental BMI at the date of the home visit, and no information about history of maternal hypoglycemia or gestational diabetes, which have been proposed as causal pathways to change DNA methylation [26].

*LEP* is a polypeptide released from adipocytes that feeds back to neurons in the hypothalamus and other brain regions to suppress appetite. The regulation of *LEP* is due, in part, to methylation at its prompter. Our finding that obese boys had lower levels of *LEP* methylation would be expected to result in higher levels of leptin in serum and appetite suppression (but rodent models have provided some results inconsistent with this finding [27]). It should be noted that changes in *LEP* promoter methylation are not restricted to adipocytes; in addition to our study on saliva, similar findings have been reported using DNA from blood samples of children [13]. Another study reported a negative correlation between *LEP* methylation, measured in blood, and BMI in 73 persons with severe obesity, although no associations were found using subcutaneous or visceral adipose tissue [28]. This absence of specificity in adipocytes suggests that promoter methylation in the *LEP* gene could be a potential biomarker for *LEP* resistance from several cell types (possibly because those other cell types, such as leukocytes, are also involved in appetite regulation).

Sex differences in the methylation levels of *LEP* have been reported previously. In a study conducted in infants (50 girls, 70 boys), higher BMI was negatively correlated with *LEP* methylation in boys [29]. In the Dutch cohort of mothers who suffered famine during War Word II, famine exposure was associated with *LEP* methylation only in the male children compared to siblings of the same sex without famine exposure (the sample size was 311 children exposed and 311 control siblings) [30]. A possible explanation for the sex differences could be due to the involvement of leptin in sexual maturation. Leptin is an important hormone in female development [31]: similar to estrogens and progesterone, serum leptin levels change across the menstrual cycle [32]. Associations between *LEP* promoter methylation and obesity in females may be difficult to discern because of the influence of sex hormones on DNA methyltransferases, the enzymes responsible for DNA methylation [33]. Prominent changes in hormone levels occur during puberty, which was likely to be occurring in many of our 10–15-year-old children.

One of the limitations of this study is that it was cross-sectional, so temporal relations of epigenetic measures and obesity-related outcomes cannot be discerned. Another limitation is that only four genes or gene sequences were evaluated, in contrast to epigenome-wide approaches, which are the current standard in epigenetic research [34]. Budget limitations precluded epigenome-wide measurements in our large sample. Finally, we did not have circulating leptin measurements, which would have been very helpful in assessing the biological plausibility of our findings.

## Conclusions

We found an association between DNA methylation in each CpG site of the gene *LEP*, measure in saliva, and obesity-related measures in 431 children aged 10–15 years old. This association differed by sex and obesity status, with a much stronger association in obese boys. No significant associations were observed in the CpG sites tested for *ICAM-1*, *CRH* and LINE-1. The results suggests that saliva may be a useful surrogate tissue for large-scale studies of epigenetics in relation to obesity, particularly in children and adolescents for whom venipuncture is an obstacle to study participation [35]. Saliva collection is non-invasive and inexpensive, and results were biologically consistent. Future studies should focus on the temporality of epigenetic changes in relation to the onset of obesity.

## Additional files

Additional file 1: Table S1. Location of the CpG sites tested in the four candidate genes and gene sequence. (DOC 37 kb)

Additional file 2: **Table S2.** Percent methylation (mean ± SD) in each CpG site, and the overall mean, of the four genes or gene sequences tested. (DOC 98 kb)

Additional file 3: **Table S3.** Adjusted^*^ associations^†^ between obesity-related measures (dependent variables) and *ICAM-1* methylation (independent variable). (DOC 76 kb)

Additional file 4: **Figure S1.** Mean *LEP* methylation (mean of four CpG sites) plotted against the three obesity-related outcomes (BMI-z, WC-z, and PBF) for boys (blue circles) and girls (red triangles). The regression lines (fitted values) by sex are also shown in the plot. The extreme value in the body mass index z-score (<−4) was confirmed and is not an erroneous value. (DOC 1239 kb)

## Abbreviations

BMI-z: Body mass index z-score
EHR: Electronic health records
PBF: Percent body fat
WC-z: Waist circumference z-score
