# Supplementary material for: Associations of LEP, CRH, ICAM-1, and LINE-1 methylation, measured in saliva, with waist circumference, body mass index, and percent body fat in mid-childhood
Source: Clin Epigenetics. 2017 Mar 29;9:29. doi: 10.1186/s13148-017-0327-5 (PMC5372250; doi:10.1186/s13148-017-0327-5)
Supplement: Supplementary file 1 — Location of the CpG sites tested in the four candidate genes and gene sequence. (DOC 37 kb) [file 13148_2017_327_MOESM1_ESM.doc]

**Additional file 1: Table S1.** Location of the CpG sites tested in the four candidate genes and gene sequence

| **Gene** | **Sequence analyzed** | **Location on the genome** | **Qiagen catalog number** |
| --- | --- | --- | --- |
| *LEP* | CTCCGGCGCGACTATGGCGT | Chr7:  128240983-128241004 | Hs_LEP_01_PM PyroMark  CpG assay (PM00129724) |
| *ICAM-1* | CCCCGGCGGCCTCCTCGCTT  CCCCTTTCGGCCTTGGCCGC  GGAGGCGT | Chr19:  10270380-10270427 | Hs_ICAM1_02_PM  PyroMark CpG assay  (PM00186711) |
| *CRH* | CGGAAGGGAGGTAGTCAGC  GCGGGT | Chr8:  66177843-66177867 | Hs_CRH_01_PM PyroMark  CpG assay (PM00036197 |
| LINE-1 | TTCGTGGTGCGTCGTT | Repetitive sequence | PyroMark Q24 CpG LINE-1 |
