# Supplementary material for: Associations of LEP, CRH, ICAM-1, and LINE-1 methylation, measured in saliva, with waist circumference, body mass index, and percent body fat in mid-childhood
Source: Clin Epigenetics. 2017 Mar 29;9:29. doi: 10.1186/s13148-017-0327-5 (PMC5372250; doi:10.1186/s13148-017-0327-5)
Supplement: Supplementary file 2 — Table S2. Percent methylation (mean ± SD) in each CpG site, and the overall mean, of the four genes or gene sequences tested. (DOC 98 kb) [file 13148_2017_327_MOESM2_ESM.doc]

**Additional file 2: Table S1.** Percent methylation (mean ± SD) in each CpG site, and the overall mean, of the four genes or gene sequences tested

|  | **All** | **Boys** | **Girls** | **P-value** |
| --- | --- | --- | --- | --- |
| ***LEP*** |  |  |  |  |
| N | 429 | 210 | 219 |  |
| CpG-1 | 21.35 ± 5.74 | 20.55 ± 5.69 | 22.12 ± 5.70 | 0.004 |
| CpG-2 | 7.43 ± 2.51 | 7.26 ± 2.48 | 7.60 ± 2.53 | 0.15 |
| CpG-3 | 12.51 ± 3.59 | 12.07 ± 3.68 | 12.93 ± 3.45 | 0.01 |
| CpG-4 | 34.39 ± 6.29 | 33.05 ± 6.65 | 35.65 ± 5.64 | < 0.0001 |
| Mean | 18.92 ± 3.89 | 18.23 ± 3.97 | 19.58 ± 3.70 | 0.0003 |
| ***ICAM-1*** |  |  |  |  |
| N | 427 | 208 | 219 |  |
| CpG-1 | 2.41 ± 1.22 | 2.45 ± 1.34 | 2.38 ± 1.09 | 0.56 |
| CpG-2 | 2.91 ± 1.45 | 2.99 ± 1.59 | 2.84 ± 1.30 | 0.28 |
| CpG-3 | 0.95 ± 0.76 | 0.99 ± 0.82 | 0.92 ± 0.70 | 0.32 |
| CpG-4 | 5.60 ± 2.05 | 5.87 ± 2.18 | 5.34 ± 1.89 | 0.008 |
| Mean | 2.97 ± 1.06 | 3.07 ± 1.15 | 2.87 ± 0.96 | 0.05 |
| ***CRH*** |  |  |  |  |
| N | 430 | 210 | 220 |  |
| CpG-1 | 5.86 ± 3.12 | 6.01 ± 3.95 | 5.72 ± 2.04 | 0.34 |
| CpG-2 | 3.21 ± 2.44 | 3.41 ± 3.22 | 3.03 ± 1.30 | 0.12 |
| CpG-3 | 4.52 ± 2.73 | 4.72 ± 3.57 | 4.33 ± 1.54 | 0.14 |
| Mean | 4.53 ± 2.48 | 4.71 ± 3.31 | 4.36 ± 1.23 | 0.15 |
| **LINE-1** |  |  |  |  |
| N | 430 | 210 | 220 |  |
| CpG-1 | 78.68 ± 4.38 | 78.71 ± 2.38 | 78.65 ± 3.73 | 0.88 |
| CpG-2 | 74.30 ± 7.83 | 73.93 ± 8.57 | 74.67 ± 4.60 | 0.10 |
| CpG-3 | 68.02 ±6.61 | 67.65 ± 6.65 | 68.38 ± 6.58 | 0.25 |
