# Supplementary material for: Associations of LEP, CRH, ICAM-1, and LINE-1 methylation, measured in saliva, with waist circumference, body mass index, and percent body fat in mid-childhood
Source: Clin Epigenetics. 2017 Mar 29;9:29. doi: 10.1186/s13148-017-0327-5 (PMC5372250; doi:10.1186/s13148-017-0327-5)
Supplement: Supplementary file 3 — Table S3. Adjusted* associations† between obesity-related measures (dependent variables) and ICAM-1 methylation (independent variable). (DOC 76 kb) [file 13148_2017_327_MOESM3_ESM.doc]

**Additional file 3: Table S3.** Adjusted^*^ associations^†^ between obesity-related measures (dependent variables) and *ICAM-1* methylation (independent variable)

| **Anthropometric**  **measure** | ***ICAM-1 location*** | **Boys**  **β (SE)** | **Girls**  **β (SE)** | **P-value Interaction** |
| --- | --- | --- | --- | --- |
| WC-z | CpG-1 | -0.127 (0.047) | 0.018 (0.057) | 0.05 |
| PBF | CpG-1 | -1.126* (0.559) | 0.587 (0.680) | 0.05 |
| BMI-z | CpG-1 | -0.106 (0.060) | 0.024 (0.073) | 0.02 |
| WC-z | CpG-2 | -0.076 (0.040) | -0.068 (0.048) | 0.10 |
| PBF | CpG-2 | -0.642 (0.474) | -0.404 (0.574) | 0.75 |
| BMI-z | CpG-2 | -0.045 (0.051) | -0.074 (0.061) | 0.71 |
| WC-z | CpG-3 | 0.015 (0.077) | 0.045 (0.089) | 0.80 |
| PBF | CpG-3 | 1.411 (0.911) | 0.828 (1.055) | 0.68 |
| BMI-z | CpG-3 | 0.053 (0.010) | -0.003 (0.113) | 0.71 |
| WC-z | CpG-4 | -0.047 (0.029) | -0.020 (0.033) | 0.53 |
| PBF | CpG-4 | -0.168 (0.345) | 0.066 (0.393) | 0.65 |
| BMI-z | CpG-4 | -0.039 (0.037) | -0.029 (0.042) | 0.87 |
| WC-z | Mean | -0.120* (0.055) | -0.040 (0.065) | 0.35 |
| PBF | Mean | -0.659 (0.654) | 0.176 (0.781) | 0.41 |
| BMI-z | Mean | -0.086 (0.070) | -0.056 (0.083) | 0.79 |

^*^ Models adjusted for age.

^†^ P-values: *** p < 0.001; ** 0.001 ≤ p < 0.01; * 0.01 ≤ p < 0.05

Abbreviations: WC-z = waist circumference z-score; PBF = percent body fat; BMI-z = body mass index z-score
