# Supplementary material for: Associations of LEP, CRH, ICAM-1, and LINE-1 methylation, measured in saliva, with waist circumference, body mass index, and percent body fat in mid-childhood
Source: Clin Epigenetics. 2017 Mar 29;9:29. doi: 10.1186/s13148-017-0327-5 (PMC5372250; doi:10.1186/s13148-017-0327-5)
Supplement: Supplementary file 4 — Figure S1. Mean LEP methylation (mean of four CpG sites) plotted against the three obesity-related outcomes (BMI-z, WC-z, and PBF) for boys (blue circles) and girls (red triangles). The regression lines (fitted values) by sex are also shown in the plot. The extreme value in the body mass index z-score (<−4) was confirmed and is not an erroneous value. (DOC 1239 kb) [file 13148_2017_327_MOESM4_ESM.doc]

**Additional file 4: Figure S1.** Mean *LEP* methylation (mean of four CpG sites) plotted against the three obesity-related outcomes (BMI-z, WC-z and PBF) for boys (blue circles) and girls (red triangles). The regression lines (fitted values) by sex are also shown in the plot. The extreme value in the body mass index z-score (< -4) was confirmed and is not an erroneous value.

***
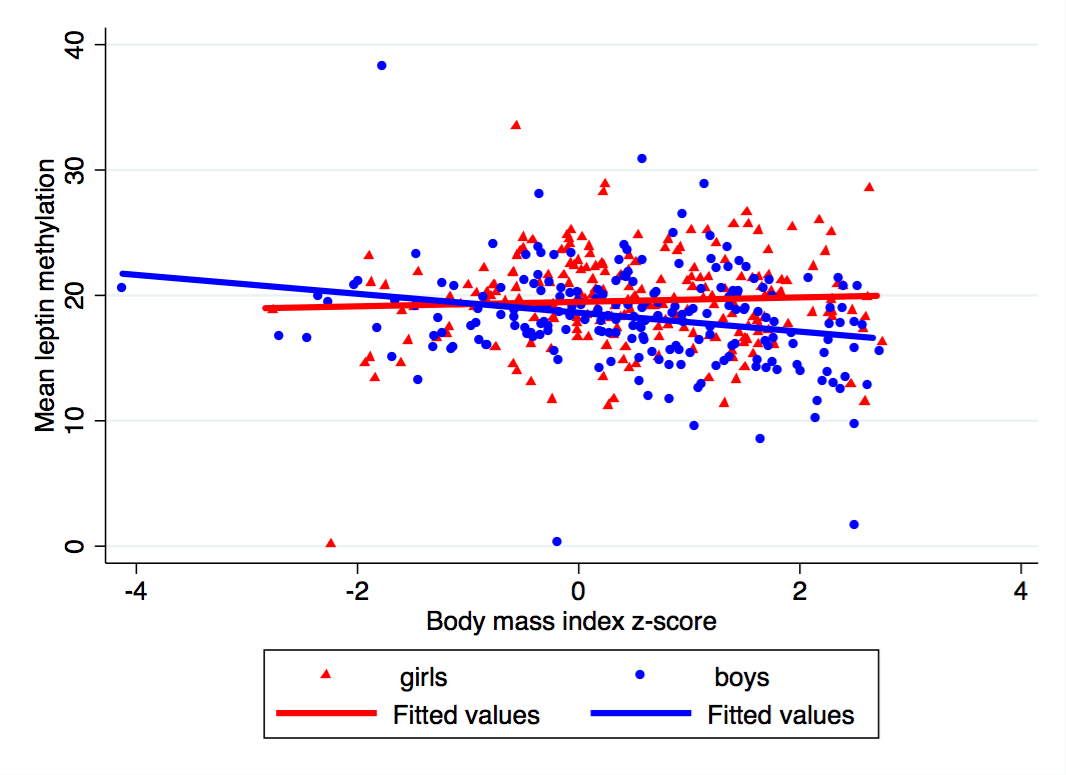
***

***
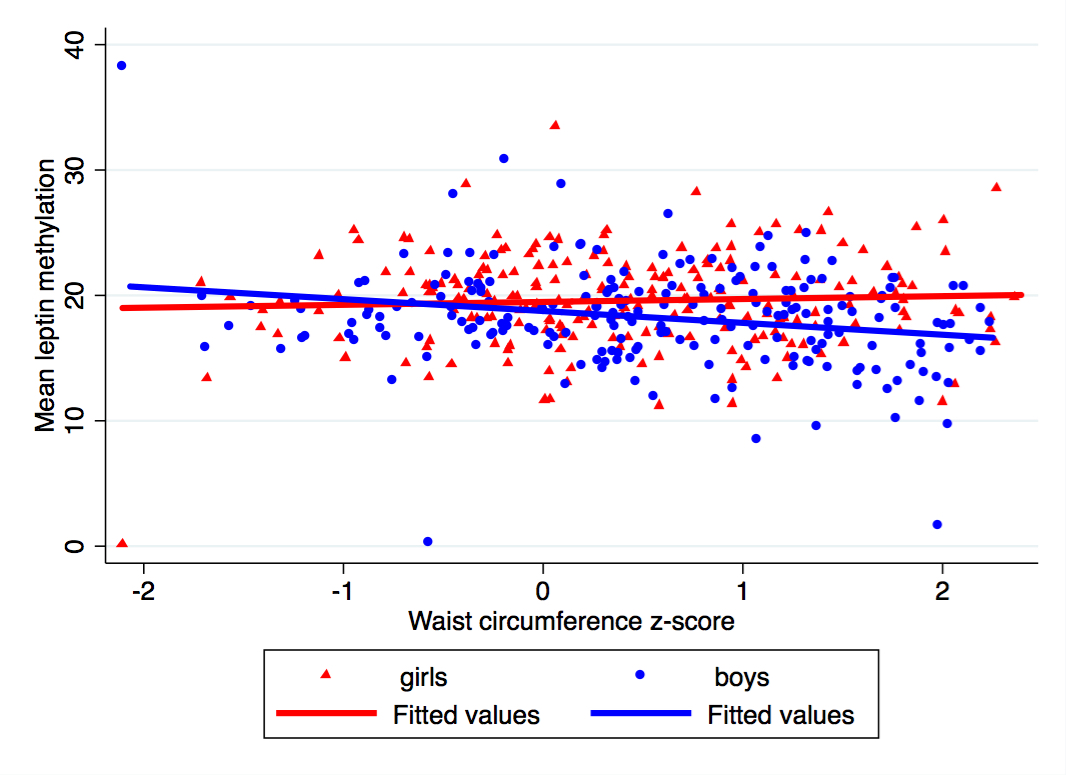
***

***
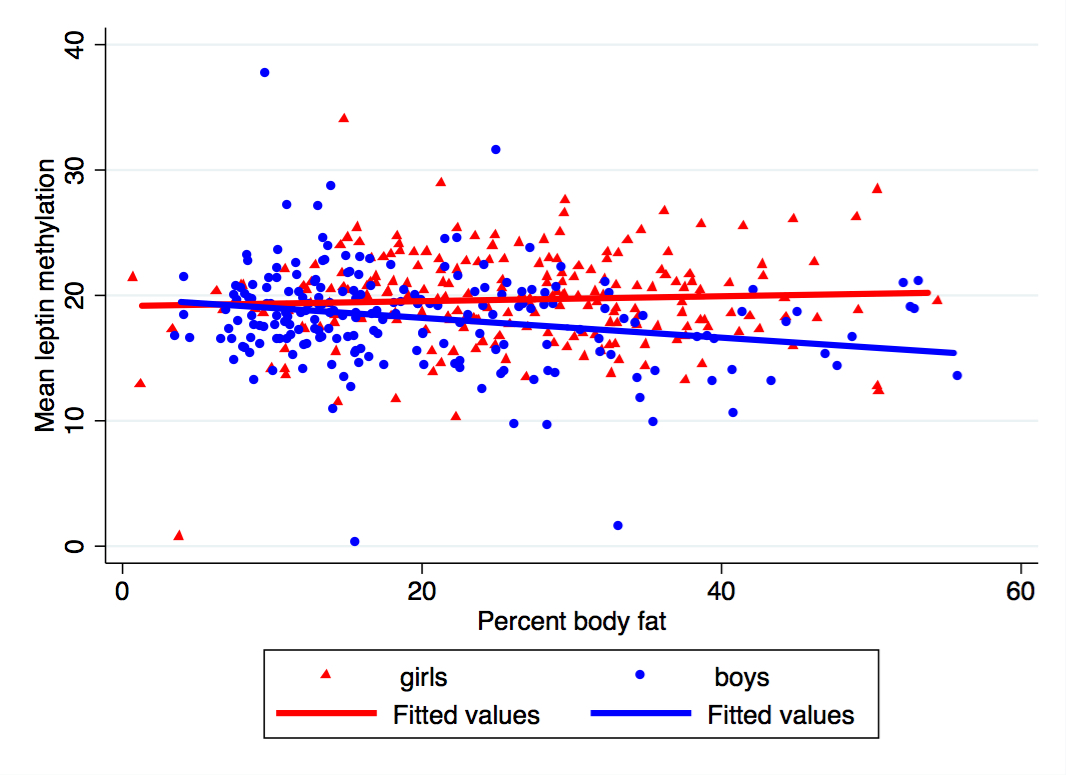
***
